# Metformin-based nanomedicines for reprogramming tumor immune microenvironment

**DOI:** 10.7150/thno.104872

**Published:** 2025-01-01

**Authors:** Jieyu Liu, Xiaoling Li, Yinggang Li, Qiyong Gong, Kui Luo

**Affiliations:** 1 Department of Radiology, Huaxi MR Research Center (HMRRC), Institution of Radiology and Medical Imaging, Breast Center, Institute of Breast Health Medicine, State Key Laboratory of Biotherapy, West China Hospital, Sichuan University, Chengdu 610041, China.; 2 Functional and Molecular Imaging Key Laboratory of Sichuan Province, NHC Key Laboratory of Transplant Engineering and Immunology, Research Unit of Psychoradiology, Chinese Academy of Medical Sciences, Chengdu 610041, China.; 3 Xiamen Key Lab of Psychoradiology and Neuromodulation, Department of Radiology, West China Xiamen Hospital of Sichuan University, Xiamen 361021, China.

**Keywords:** Metformin, AMPK, Tumor immunity, Tumor microenvironment, Nanomedicine

## Abstract

Immunotherapy has transformed current cancer management, and it has achieved significant progress over last decades. However, an immunosuppressive tumor microenvironment (TME) diminishes the effectiveness of immunotherapy by suppressing the activity of immune cells and facilitating tumor immune-evasion. Adenosine monophosphate-activated protein kinase (AMPK), a key modulator of cellular energy metabolism and homeostasis, has gained growing attention in anti-tumor immunity. Metformin is usually considered as a cornerstone in diabetes management, and its role in activating the AMPK pathway has also been extensively explored in cancer therapy although the findings on its role remain inconsistent. Metformin in a nanomedicine formulation has been found to hold potential in reprogramming the immunosuppressive TME through immunometabolic modulation of both tumor and immune cells. This review elaborates the foundation and progress of immunometabolic reprogramming of the TME via metformin-based nanomedicines, offering valuable insights for the next generation of cancer therapy.

## Introduction

Over the past decades, extensive studies have gradually unveiled the characteristics of the tumor microenvironment (TME), which is considered as an evolving entity composed of heterogeneous cell populations, abnormal vasculature, cytokines and extracellular matrix (ECM) 1. There are various cell populations within the TME, including immune cells, tumor cells and stromal cells, and the specific proportion of each cell type varies in different cancer types 2. The ECM is a non-cell component primarily secreted by cancer-associated fibroblasts (CAFs), and it serves as a reservoir for various immunosuppressive cytokines and growth factors 3. Studies have indicated that the heterogeneous TME supports tumor progression and impedes immunotherapeutic action from multiple aspects. (I) A high interstitial pressure and a dense ECM within the TME are two physical barriers to block deep penetration of drugs and immune cells 4; (II) Various suppressive cytokines, which are secreted by tumor cells and immunosuppressive cells such as tumor-associated macrophages (TAMs), regulatory T (T_reg_) cells and myeloid derived suppressor cells (MDSCs), facilitate tumor immunoevasion 5; (III) Overexpressed immune checkpoint molecules suppress T cell function and proliferation 6; (IV) Metabolic stress impairs anti-tumor immunity by depriving essential nutrients and allowing suppressive metabolite accumulation, leading to the exhaustion of cytotoxic T lymphocytes (CTLs) 7 and the proliferation of TAMs 8 and T_reg_ cells 9. It is noted that metabolic reprogramming of the TME has become as a novel approach to enhancing anti-tumor immunotherapeutic effects 10-15.

Metformin is commonly regarded as the primary medication for individuals with type 2 diabetes mellitus (T2DM) due to its proven ability to lower the blood sugar level as well as improve insulin sensitivity. In 1922, metformin was first synthesized, and its blood glucose-lowering function was confirmed in rabbits by 1929 16. Finally in 1994, metformin was approved by the Food and Drug Administration (FDA) and it is currently widely used in clinical practice to treat T2DM. The most acknowledged anti-diabetic mechanisms of metformin involve in reducing hepatic gluconeogenesis and increasing intestinal secretion of glucagon-like peptide 1 (GLP1) through activating the adenosine monophosphate-activated protein kinase (AMPK) signaling pathway 17, 18. The extensively studied phosphatidylinositol-3-kinase (PI3K) and mitogen-activated protein kinase (MAPK) signaling pathways are critical regulatory hubs for cell metabolism, survival, and proliferation 19-21. Thus, inhibitors of PI3K and rat sarcoma (RAS) are considered as highly specific medicines approved to treat patients with cancer 22, 23. Interestingly, while the AMPK pathway is essential for maintaining cellular homeostasis, its tumor-suppressive mechanism remains unclear. Abundant clinical evidence suggests that diabetes occur frequently in patients with many kinds of cancer, indicating metformin as an AMPK pathway activator is a potential adjuvant in cancer management 24. Emerging evidence shows that metformin-mediated AMPK activation is closely linked to its anti-tumor immune response. On the one hand, it directly suppresses tumor growth through inhibiting various anabolic processes, on the other hand, it reprograms the immunosuppressive TME by increasing the metabolic fitness of inflammatory immune cells 25, 26. Hence, metformin holds promise as an adjuvant of cancer treatment, extending its benefits beyond diabetes management.

The poor pharmacokinetics and bioavailability of metformin hamper its clinical application in cancer therapy. Advances in nanotechnology promote the development of nanomedicines, which are a nano-formulation of therapeutic drugs including metformin. Nanomedicines have played an impactful role in the metabolic reprogramming approach 27. Upon intravenous administration, vascular endothelial gaps and impaired lymphatic drainage allow nanomedicines to concentrate within tumor sites through the enhanced permeability and retention (EPR) effect 28. Commonly overexpressed receptors on target cells can be utilized in preparing nanomedicines to target the cell type of interest via receptor-ligand interaction, thus achieving high-efficiency delivery of therapeutic drugs into tumor tissues and revitalizing anti-tumor immune response 29, 30. Besides, multiple therapeutic drugs can be integrated into one nanomedicine to induce immunogenic cell death (ICD) via different treatment modalities. For instance, photothermal therapy (PTT) can damage tumor cells through local heat, while chemodynamic therapy (CDT) and photodynamic therapy (PDT) generate reactive oxygen species (ROS) to induce tumor cell apoptosis 31. Impaired or dead tumor cells release damage-associated molecular patterns (DAMPs) including ATP, calreticulin (CRT) and high mobility group box 1 (HMGB1) to activate dendritic cells (DCs) and subsequent T cells for anti-tumor immunity initiation 32. Chemotherapy and radiotherapy can also induce ICD to recruit lymphocytes to initiate the tumor-immunity cycle, creating an immunologically hot TME 33, 34. Therefore, a myriad of therapeutic drugs for different treatment modalities have been incorporated into metformin-containing nanomedicines, which have shown remarkable effects on tumor regression.

In this review, we first introduce the basic structure and function of the AMPK complex, and then delve into the anti-tumor mechanisms of metformin by targeting the AMPK pathway in tumor cells, T cells and TAMs. We examine the efficacy of metformin in its current clinical use for cancer treatment, and demonstrate clinical benefits of metformin-containing nanomedicines in comparison with free metformin. Finally, we offer prospective insights into reprogramming the TME via metformin, which could help transition of metformin-containing nanomedicines to clinical use in cancer therapy. Given that metformin exerts anti-tumor effects predominately through inhibition of oxidative phosphorylation (OXPHOS) and AMPK-dependent manner, this review specifically focuses on the impact of above two mechanisms on immunometabolic modulation, excluding other AMPK-independent anti-tumor mechanisms of metformin such as increasing reactive oxygen (ROS) and inhibiting the oncogenic wingless-related integration site (Wnt) pathway 35.

## Metformin reprograms the tumor microenvironment through activating the AMPK pathway

The AMPK trimer, a critical regulator of cellular energy metabolism, consists of one catalytic α subunit and two regulatory β and γ subunits, each with different isoforms. For instance, the α and β subunits have two subtypes, while the γ subunit has three 36. The heterotrimeric structure of AMPK allows for coordinated and intricate energy-regulating function, and regulatory β and γ subunits play roles in maintaining AMPK complex stability and binding to AMP as an AMPK stimulator, respectively 37. Metformin-mediated mitochondrial complex I inhibition reduces ATP production and induces energy stress, resulting in an elevation in the AMP level. After AMP binds to the γ subunit, upstream liver kinase B1 (LKB1) is activated to phosphorylate Thr172 on the α subunit, thereby activating the AMPK complex 38. Additionally, an increased level of intracellular Ca^2+^ activates the AMPK pathway via calcium/calmodulin-dependent kinase kinase (CaMKK2), which is a non-canonical, LKB1-independent mechanism of AMPK activation 39.

The AMPK signaling pathway is a central regulator of energy metabolism in eukaryotes. AMPK activation typically inhibits the mTOR activity, thereby suppressing biosynthetic processes for proteins and lipids, while enhancing breakdown processes such as fatty acid oxidation (FAO) 40. In addition, AMPK activation promotes autophagy and mitophagy via unc51-like kinase-1 (ULK1) phosphorylation to control the mitochondrial mass 41. Mitochondrial biogenesis can also be promoted by AMPK activation via phosphorylating peroxisome proliferator activated receptor γ coactivator-1α (PGC-1α), a transcriptional coactivator, and an increase in the mitochondrial mass helps coping with intracellular energy stress 42.

Although metformin is well known as the first-line treatment drug for T2DM, recent clinical trial studies have demonstrated its potential in cancer therapy 43, 44. Accumulating evidence suggests that metformin as an AMPK activator can boost anti-tumor immune response and inhibit tumor progression 45, 46. For instance, the metformin-activated AMPK signaling pathway regulates the TME by reducing programmed cell death 1 ligand 1 (PD-L1) expression and inhibiting anabolic metabolism in tumor cells, repolarizing TAMs, and improving T cell metabolic fitness. In this section, we elaborate immunometabolic modulation in different cell types within the TME by metformin and reveal its tumor-suppressing effects as evidenced by recent clinical trial findings.

### Metformin suppresses anabolic metabolism and PD-L1 expression in tumor cells

Tumor cells alter their metabolic profiles and rely on aerobic glycolysis more than OXPHOS even in the presence of sufficient oxygen, because aerobic glycolysis generates a great number of intermediate metabolites for biosynthesis of proteins, lipids and nucleotides, which is referred to the “Warburg effect” 44. The PI3K- protein kinase B (AKT)-mammalian target of rapamycin (mTOR) axis is frequently hyperactivated during tumor progression, which can induce the overexpression of hexokinase 2 (HK2) and glucose transporter 1 (GLUT1), thereby sustaining a high glycolysis flux 47. The PI3K and AMPK signaling pathways typically exert an opposing effect on tumor metabolism. Metformin-mediated AMPK activation can inhibit the mammalian target of rapamycin complex 1 (mTORC1) activity via phosphorylating Raptor as a scaffolding protein and tuberous sclerosis complex 2 (TSC2) as a negative regulator, thereby suppressing various anabolic processes in tumor cells 48. Inhibition of mTORC1 downregulates the expression of its downstream targets, such as hypoxia inducible factor-1α (HIF-1α), ribosomal protein S6 kinase (S6K) and eukaryotic initiation factor 4E (eIF4E), thereby blocking glycolysis and preventing protein translation 49. Apart from glucose and amino acid metabolism, lipid metabolism is crucial for cell growth 50. Since fatty acids and cholesterol are vital for stabilizing cell membranes, fatty acid synthesis (FAS) and cholesterol production are often upregulated in tumor cells 51. AMPK activation downregulates the expression of sterol regulatory element-binding protein 1 (SREBP1), a key transcriptional regulator for fatty acids and cholesterol metabolism 52. Besides, acetyl-CoA carboxylase (ACC) is a key regulator for FAS, which facilitates the transformation of acetyl-CoA into malonyl-CoA for subsequent synthesis reactions. AMPK activation can phosphorylate ACC1 and ACC2, thus inhibiting their activity and lowering the FAS level 53. It has been shown that AICAR as an AMPK activator could decrease the cholesterol level through downregulating the HMG-CoA reductase (HMGCR) pathway 54. Therefore, AMPK activation can inhibit anabolic metabolism for synthesis of amino acids and lipids that are critical to tumor cell growth, suggesting metformin-mediated AMPK activation has the potential to reprogram tumor metabolism and enhance immunometabolic therapeutic effects.

In addition to disrupting tumor cell metabolism, metformin has been reported to promote proteasomal degradation of PD-L1. One of predominant immunosuppressive mechanisms in tumor cells is overexpression of immune checkpoint molecules. For instance, upregulated PD-L1 on tumor cells typically engages with programmed cell death 1 (PD-1) on effector T (T_eff_) cells, leading to a reduction in effector cytokine secretion and inducing apoptosis of T_eff_ cells 55. An array of immune checkpoint inhibitors (ICIs) has been developed to mitigate PD-L1/PD-1-mediated immunosuppression, but their therapeutic outcomes are suboptimal and their application is accompanied with severe side effects 56. Interestingly, AMPK activation induces abnormal glycosylation of PD-L1, leading to its translocation from endoplasmic reticulum to cytoplasmic proteasomes, where mis-aligned proteins undergo degradation 57. Therefore, metformin may hold great potential in disrupting the immunosuppressive signaling from PD-L1 and restoring tumoricidal ability of T_eff_ cells.

Hypoxia is considered as a prevalent characteristic of solid cancers. Rapid tumor cell proliferation drives abnormal angiogenesis and the resulting disorganized vasculature prevents oxygen diffusion into distal blood vessels, resulting in a hypoxic TME that contributes to tumor progression 58. Hypoxia facilitates tumor immune evasion by impairing both innate and adaptive anti-tumor immunity. Specifically, under hypoxia stress, tumor cells secrete interleukin-10 (IL-10) and transforming growth factor-β (TGF-β) to drive M2-TAM polarization 59, and they upregulate the expression of CD39 and CD73 to generate adenosine as a toxic metabolite, which diminishes CD8^+^ T cell cytotoxicity 60. Notably, hypoxia also decreases HIF-1α degradation and upregulates the PD-L1 expression in the TME, thus facilitating tumor immunoevasion 61. Metformin could be applied to alleviate hypoxia in the TME via inhibiting complex I and mitochondrial respiration in tumor cells, thus mitigating hypoxia-induced immunosuppression 62. An elevated oxygen concentration also enhances the efficacy of radiotherapy and PDT because both therapies are highly dependent on cytotoxic ROS generated from oxygen.

**Figure 1:**
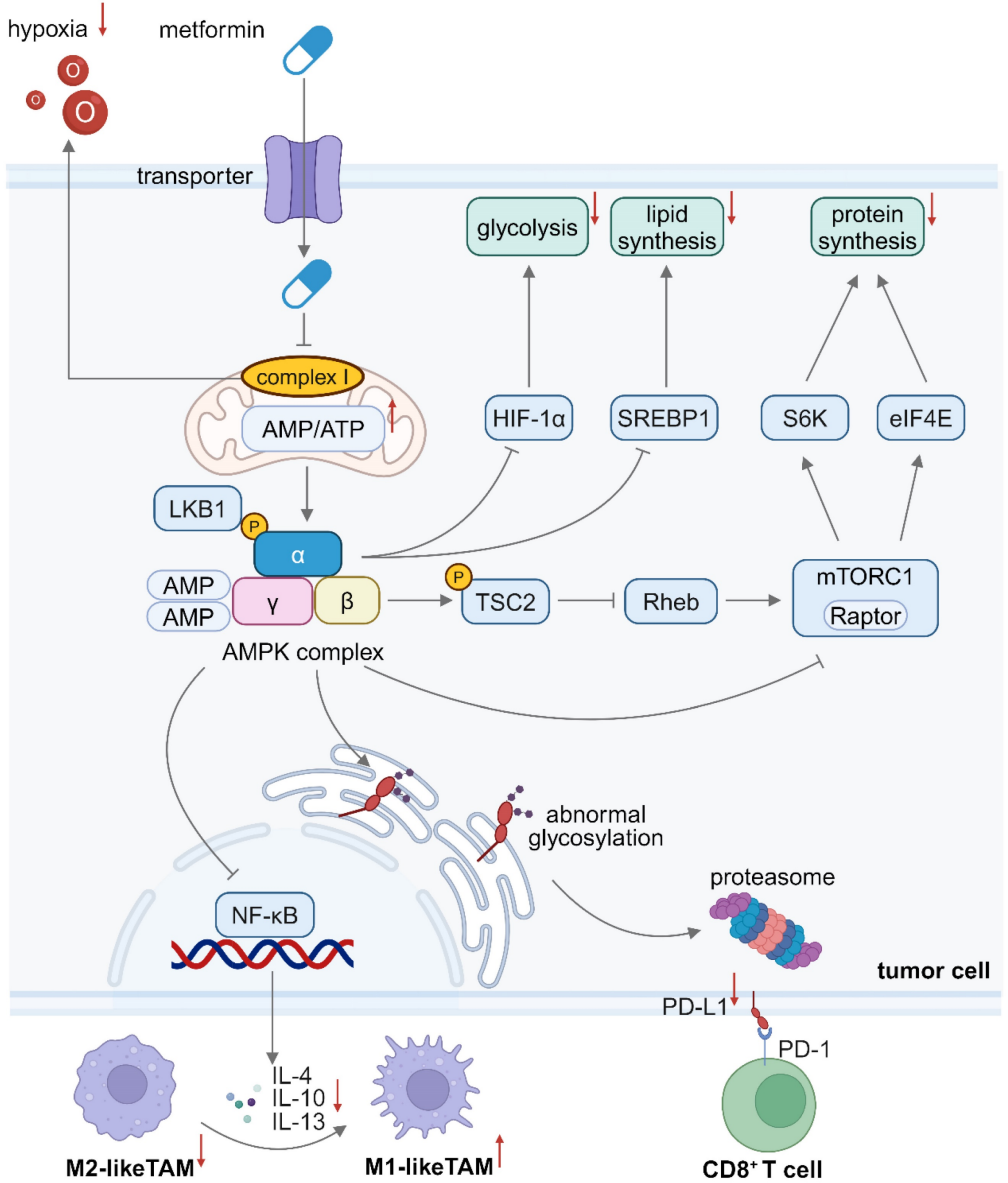
Schematic illustration of the mechanism of inhibiting tumor cell growth and reprogramming the TME by metformin. Metformin inhibits mitochondrial complex I, reducing oxygen consumption in tumor cells and alleviating hypoxia in the TME. Metformin-induced AMPK activation leads to mTORC1 inhibition in tumor cells, thus reducing glycolysis, protein synthesis and lipid synthesis. The AMPK pathway activation in tumor cells also inhibits the secretion of immunosuppressive cytokines, which facilitates M1-to-M2 polarization of TAMs. Besides, AMPK activation in tumor cells induces abnormal glycosylation and proteasomal degradation of PD-L1 proteins. Downregulated PD-L1 expression on tumor cells weakens the immunosuppressive signal from PD-L1/PD-1 engagement, thus enhancing CD8^+^ T cell cytotoxicity. Created in https://BioRender.com. AMPK: adenosine monophosphate-activated protein kinase; eIF4E: eukaryotic initiation factor 4E; HIF-1α: hypoxia inducible factor-1α; LKB1: liver kinase B1; mTORC1: mammalian target of rapamycin complex 1; NF-κB: nuclear factor kappa-B; PD-L1: programmed cell death 1 ligand 1; PD-1: programmed cell death 1; S6K: ribosomal protein S6 kinase; SREBP: sterol regulatory element-binding protein; TME: tumor microenvironment.

In conclusion, metformin contributes to creation of an immunogenic TME via several approaches. The metformin-activated AMPK signaling pathway reprograms metabolic processes of tumor cells, including protein and lipid biosynthesis via mTOR inhibition, thereby inhibiting tumor growth. Metformin also downregulates PD-L1 expression and ameliorates hypoxia within the TME, fostering a more immunogenic milieu against tumor immune evasion (**Figure 1**). Furthermore, an increase in the oxygen level in the TME enhances the efficacy of oxygen-dependent treatment modalities.

### Metformin enhances mitochondrial biogenesis and memory differentiation of T cells

During tumor regression, T cells and their metabolic fitness are crucial for effective anti-tumor immunity. Importantly, the T cell metabolic profile changes dynamically during their development course. For example, naïve T cells exhibit a resting metabolic phenotype and they harness OXPHOS to produce ATP. Upon stimulation by tumor antigens and co-stimulatory molecules, glycolysis is significantly upregulated in naïve T cells and they become activated. Activated T_eff_ cells are the major tumor-killing immune cells, and they exhibit an elevated level of glycolysis and OXPHOS 63. After curbing tumor growth, the majority of T_eff_ cells perish through apoptosis while a small subset survives and transforms into memory T (T_m_) cells that respond to future encounters with tumor antigens for a long time 64. Unfortunately, T cells can not compete with tumor cells for nutrients, resulting in an immunosuppressive TME. For example, glucose and glutamine deficiencies inhibit the activation of T_eff_ cells and prevent interferon-γ (IFN-γ) production from them 7. In addition, T_m_ cells predominately perform OXPHOS and fatty acid oxidation (FAO) to maintain self-renewal and cope with metabolic stress 56. The AMPK signaling pathway serves as an energy sensor in glucose-starved T_eff_ cells, and it helps restoring a quiescent metabolic profile and facilitates T_m_ cell generation. Therefore, promoting memory immunity of T cells through activating the AMPK pathway offers a promising strategy to enhance anti-tumor immune response.

The precise impact of AMPK activation on T cell immunity remains to be unveiled. It is known that metformin activates AMPK to downregulate glycolysis in tumor cells partially through inhibiting mTORC1 and HIF-1α, and it may impair glycolysis in T_eff_ cells in a similar manner, weakening their anti-tumor immune response. Nevertheless, AMPK is crucial for T_eff_ cells to mount a rapid secondary immune response, as their differentiation into T_m_ cells entails a metabolic transition of hyperactive glycolysis to quiescent OXPHOS 65. Although glucose-derived acetyl-CoA is the primary fuel for the tricarboxylic acid (TCA) cycle, metformin-treated CAR-T cells upregulate acyl-coA synthetase short-chain family member 1 (ACSS1) to produce acetyl-CoA from acetate to fuel the TCA cycle 66, enhancing their proliferation and tumor-killing capability through effective OXPHOS and energy production 67.

Beyond OXPHOS, FAO is essential for T_m_ cell formation and tumor immunosurveillance. TNF receptor-associated factor 6 (TRAF6) promotes T_m_ cell differentiation by upregulating FAO. AMPK activation and memory formation are impaired in TRAF6-deficient T cells, while they can be restored after metformin interventions, suggesting that metformin promotes T_m_ cell generation via AMPK activation and FAO 68.

FAO and fatty acid synthesis (FAS) typically have opposite effects. Malonyl-CoA, an intermediate metabolite produced during FAS, can inhibit carnitine palmitoyl-transferase 1A (CPT1A) as a crucial enzyme involved in FAO 69. AMPK activation induces inhibitory phosphorylation of Ser79 and Ser212 on two key FAS enzymes ACC1 and ACC2, respectively, thereby promoting FAO 70. Interestingly, unlike T_eff_ cells, T_m_ cells primarily synthesize endogenous fatty acids via glycolysis rather than engulfing exogenous fatty acids via CD36. The synthesized lipids are broken down in lysosomes to produce free fatty acids for FAO in the mitochondria. Although this is an inefficient cycle for lipid metabolism in T_m_ cells, primed fatty acids may stimulate T_m_ cells for rapid reactivation 71. Moreover, metformin has been reported to promote lysosomal lipolysis and mitochondrial FAO, indicating it may play a role in supporting T_m_ cell development 72. It is well established that T_eff_ cell expansion primarily relies on glycolysis for ATP production, and their transition to quiescent T_m_ cells triggers a metabolic shift. However, the precise role of metformin in regulating T_m_ cell differentiation and enhancing anti-tumor immunity remains to be elucidated.

Since OXPHOS and FAO occur primarily in the mitochondria, the mitochondrial fitness of immune cells is crucial against tumor progression. Dysfunctional tumor-infiltrating cells (TILs) often exhibit a metabolic disorder due to an insufficient mitochondrial mass. The loss of mitochondrial function in TILs has been ascribed to chronic tumor antigen stimulation, which persistently activates the AKT signaling pathway and suppresses the forkhead box O (Foxo)-PGC1α axis, impairing PGC1α-mediated mitochondrial biogenesis 73. AMPK activation can enhance the PGC1α activity to promote mitochondrial biogenesis and OXPHOS in T cells, thereby supporting long-term anti-tumor immunity 74. Beyond mitochondrial dysfunction, PD-1 on T cells interacting with PD-L1 within the TME also induces T cell exhaustion. Downregulation of PD-1 on T cells offers an alternative strategy for restoring anti-tumor immunity. It has been found that AMPK activation downregulates miR-107, a negative regulator for a transcription factor eomesodermin (Eomes), which is crucial for T_m_ cell differentiation and PD-1 expression reduction 75. Thus, metformin-mediated AMPK activation promotes T_m_ cell formation and downregulates PD-1 expression on their surfaces, contributing to a sustained tumor immunosurveillance.

**Figure 2:**
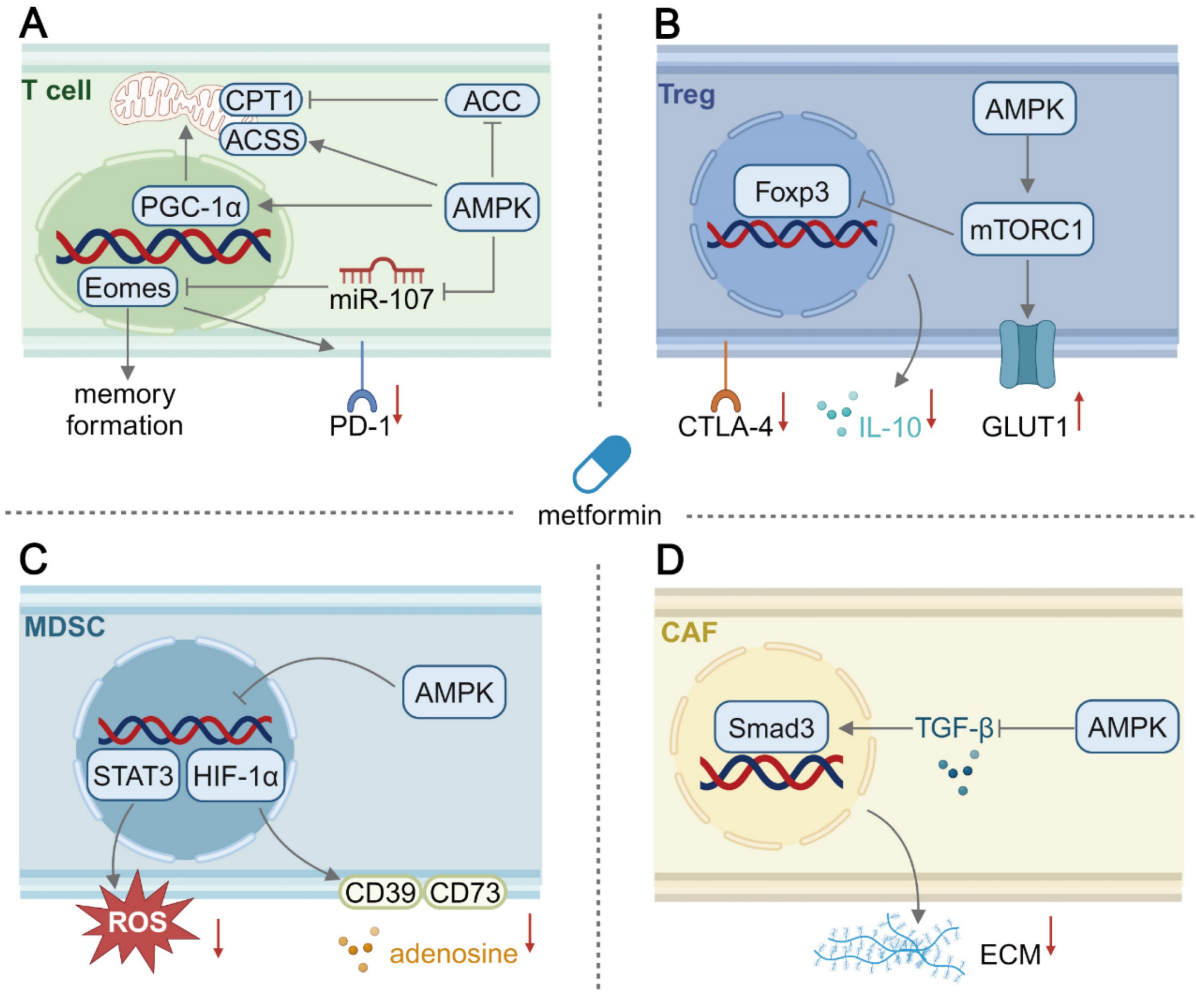
Schematic illustration of the mechanism of immunometabolic reprogramming of other cells in the TME by metformin. (A) In T cells, activation of the AMPK pathway promotes mitochondrial biogenesis and upregulates the FAO level. Besides, the AMPK pathway activation leads to memory differentiation and reduced PD-1 expression in T cells. (B) In T_reg_ cells, AMPK activation surprisingly upregulates glycolysis, inhibiting inducible T_reg_ differentiation and their CTLA-4 expression and IL-10 production. (C) In MDSCs, the production of suppressive adenosine and ROS secretion can be downregulated by metformin-mediated AMPK activation. (D) In CAFs, AMPK activation could suppress profibrotic signaling and reduce extracellular matrix deposition. Created in https://BioRender.com. ACC: acetyl-coA carboxylase; ACSS1: acyl-coA synthetase short-chain family member 1; CPT1: carnitine palmitoyltransferase 1; CTLA-4: cytotoxic T lymphocyte associate protein-4; ECM: extracellular matrix; Eomes: eomesodermin; Foxp3: forkhead box p3; GLUT1: glucose transporter 1; PGC-1α: peroxisome proliferator activated receptor γ coactivator-1α; STAT3: signal transducer and activator of transcription 3; TGF-β: transforming growth factor-β.

Studies have shown that inhibiting AKT and glycolysis can enhance FAO in T_m_ cells and improve anti-tumor immune responses 76. Metformin-mediated AMPK activation in T cells is considered as a promising approach to enhancing metabolic adaptation and promoting their memory immune response formation (**Figure 2**). Moreover, maintenance in mitochondrial fitness and mitigation in T cells exhaustion by metformin support long-term anti-tumor immunity within an immunosuppressive TME.

### Metformin-induced repolarization of tumor-associated macrophages: shifting toward an anti-tumor phenotype

TAMs, innate immune cells within the TME, exhibit heterogeneous metabolic preferences and play distinct roles in tumor progression. M1-like TAMs primarily rely on glycolysis and exert anti-tumor effects by secreting inflammatory cytokines including IL-12 and nitric oxide (NO). In contrast, M2-like TAMs prefer OXPHOS and FAO, and they inhibit T cell tumoricidal activity and support tumor progression through releasing immunosuppressive cytokines including IL-10 and IL-4 77.

In a recent clinical trial using metformin to treat esophageal squamous cell carcinoma, the infiltration of tumor-suppressing macrophages was evaluated, and the result suggested that metformin could induce the M2-to-M1 repolarization of TAMs 44. Metformin treatment inhibited mitochondrial complex I and decreased the respiratory capacity, leading to adaptive upregulation of glycolysis to maintain energy production. Such a metabolic shift from OXPHOS to glycolysis triggered the repolarization of M2-like TAMs to M1-like, thus rewriting the immunosuppressive TME 78. Therefore, metformin could be harnessed to repolarize TAMs and mitigate an immunosuppressive TME through triggering the metabolic shift from OXPHOS to glycolysis.

However, the influence of AMPK activation on macrophage polarization is complex and sometimes contradictory. For example, acadesine (AICAR), an AMPK activator, suppresses inflammatory response by inhibiting TNF-α secretion from M1-like macrophages 79, and the AMPK α1 subunit is critical in inducing the conversion of macrophages into an anti-inflammatory M2-like phenotype via IL-10 80. In diabetic cardiomyopathy, metformin is reported to induce the differentiation of M2-like macrophages by inhibition of mTOR and NLRP3 inflammasomes through AMPK activation 81, which indicates AMPK activation can alleviate excessive inflammation in autoimmune diseases. Interestingly, AMPK activation may play an anti-tumor role via repolarization of M2-like macrophages. Immunosuppressive IL-4, IL-10 and IL-13 can induce polarization of macrophages to a M2-like phenotype, while metformin-treated cancer cells exhibit a decrease in the secretion of these immunosuppressive cytokines by activating AMPK and downregulating nuclear factor kappa-B (NF-κB) p65 phosphorylation 82. Additionally, activation of the AMPK α1 subunit in macrophages is shown to play an essential role in inhibiting M2 phenotype polarization induced by IL-13 83. In this context, direct effects of metformin treatment on macrophage polarization are still under debate. The role of AMPK activation in macrophage polarization may be highly dependent on interactions between cancer cells and macrophages under different pathophysiological conditions, and these interactions remain to be unveiled.

### Metformin-mediated immunometabolic regulation in other cells

The effects of metformin may be exerted on other cell types within the TME (**Figure 2**), such as T_reg_ cells, MDSCs 84 and CAFs 85. T_reg_ cell, a subset of CD4^+^ T cells, can prevent autoimmune conditions and promote tumor progression by suppressing CTLs. The metformin-activated AMPK signaling pathway has been reported to inhibit tumor cell growth via inhibiting mTOR 86, which could be applied to T_reg_ cells. Indeed, AMPK activation has been reported to sustain T_reg_ cell function via inhibiting mTOR and upregulating FAO in T1DM 87. It was surprisingly found from one study that metformin treatment reduced the generation of tumor-infiltrating T_reg_ cells *in vitro* through activating AMPK and subsequent mTOR signaling, and an elevated glycolysis/OXPHOS ratio was found to contribute to a decrease in the expression of the master transcription factor forkhead box protein P3 (Foxp3) 88. AMPK activation to inhibit or activate mTOR in T_reg_ cells should be investigated both *in vitro* and *in vivo*. MDSCs originate from hematopoietic stem cells, and their differentiation into mature myeloid cells is often blocked during tumor progression. Immature MDSCs contribute to an immunosuppressive TME via producing a variety of suppressive cytokines and metabolites 89. Inhibiting the immunosuppressive activity of MDSCs represents an effective approach to reprogramming the TME. AMPK activation in MDSCs induced by metformin inhibits the signal transducer and activator of transcription 3 (STAT3) signaling pathway and its downstream ROS production, thus reducing their suppressive effect on CD4^+^ T cells 90. In addition, metformin interventions result in AMPK phosphorylation to inhibit HIF-1α, thus reducing the expression of CD39 and CD73 on MDSCs and decreasing adenosine production, ultimately forming an improved immune-supportive TME 91. Interestingly, in another study, the AMPK α1 subunit helped maintaining the immunosuppressive activity of MDSCs, while AMPK-deficient MDSCs exhibited tumoricidal activity by producing cytotoxic NO 92. Similar to T_reg_ cells, the role of metformin-mediated AMPK activation in modulating immune response of MDSCs is controversial, and the effects of AMPK activation may be distinguishable by investigating different subtypes of MDSCs. CAFs secrete major components of the dense ECM to form a physical barrier for infiltration of immune cells and therapeutics 93, thus a strategy could be developed to reduce ECM formation and improve an immunosuppressive TME. In stroma-rich pancreatic ductal adenocarcinoma, the dense stroma could be effectively disrupted by metformin because it activated the AMPK pathway and inhibited the secretion of profibrogenic TGF-β to reduce ECM protein secretion by stellate cells 94. Consequently, after metformin treatment, the dense ECM became thinner to allow the penetration of a gemcitabine-loaded nanomedicine 95. In conclusion, metformin could act as a potent cancer therapy adjuvant, but its effects on immunometabolic modulation in other cell types within the TME remain to be unveiled through thorough investigations.

## Lessons learned from clinical trials

The use of metformin as an adjuvant in cancer treatment has been widely explored for various cancer types (**Table 1**). The most recognized anti-tumor mechanism of metformin is its activation of the AMPK signaling pathway, which in turn inhibits the typically upregulated PI3K/AKT/mTOR pathway involved in tumor progression 96. Melanoma often displays encouraging immunogenicity, indicating its favorable response to ICI-based immunotherapy 97. Disrupting the PD-1/PD-L1 pathway is conducive to inducing objective response in advanced melanoma, and the combination of metformin treatment and immunotherapy could be particularly promising 98, 99. However, a randomized controlled phase III clinical trial found that metformin did not enhance the efficacy of pembrolizumab in resected melanoma, and recurrence-free survival (RFS) was not significantly extended in the pembrolizumab-treated group. Notably, although metformin is the first-line prescribed drug for T2DM, cancer patients with T2DM generally experienced the worse RFS in comparison with those without T2DM 100. Fortunately, there was no evidence to show that metformin treatment worsened the prognosis in melanoma patients, suggesting metformin treatment of melanoma is quite safe but its clinical benefits may be limited 101. A more recent study reported that metformin prolonged the overall survival (OS) rather than the cancer specific survival (CSS) in individuals with cutaneous melanoma. The positive effect was dose-dependent, indicating that metformin benefited melanoma patients through indirect comorbidity control rather than direct cancer prevention 102. Colorectal cancer often begins with precancerous conditions such as hyperplastic polyps or adenomas, thus polypectomy could be used as an effective preventive measure 103. In one trial, the use of metformin was evaluated in post-polypectomy patients without diabetes. Long-term administration of metformin (250 mg/day) inhibited neoplasia or polyp recurrence, suggesting mTOR inhibition mediated by metformin could suppress protein biosynthesis and proliferation of tumor cells 104. Breast cancer ranks as the second most prevalent cancer affecting women globally, and its incidence continues to rise 105. The expression level of estrogen and progesterone receptors is well correlated with breast cancer prognosis, while approximately 10-15% of breast cancer patients lack both hormone receptors and human epidermal growth factor receptor 2 (HER2), and this breast cancer is classified as triple-negative breast cancer. A significant number of breast cancer patients are resistant to immunotherapy, thus the combination of immunotherapy with other treatment modalities could be advantageous in treating breast cancer 106. A phase III clinical trial result indicated that metformin failed to improve the disease-free survival (DFS) for invasive breast cancer patients without diabetes, suggesting metformin as an adjuvant agent may be insufficient to enhance anti-tumor immune response in breast cancer treatment 107.

Although metformin has been reported to lower cancer risks and improve patient outcomes, these results remain controversial and the anti-tumor effects of metformin may be diminished when confounding factors are accounted for 114. One of the predominant factors is insufficient metformin accumulation in tumor sites. It is important to note that metformin has been reported to accumulate preferentially in the liver and gastrointestinal system rather than in tumor tissues, thus hepatic and intestinal uptake of orally administered metformin often results in poor systemic biodistribution and low bioavailability 115. Interestingly, it has been recently reported that the plasma concentration of metformin in breast cancer patients was at a micromolar level, whereas an effective anti-tumor effect through inhibition of complex I by metformin requires a dose at a millimolar level 116. It is also confirmed that high-dose metformin exhibits direct anti-tumor effect *in vitro* 117, but oral administration of metformin at a high dose may cause severe side effects, such as lactic acidosis 118. These findings suggest that targeted delivery of metformin to tumor sites could resolve challenging issues associated with traditional oral administration.

The use of metformin has been explored in many preclinical and clinical cancer studies, while outcomes from these studies are controversial. Oral administration of metformin could be one of contributors for the controversial outcomes since there are challenging issues, such as unspecific biodistribution, poor tumor accumulation and non-selective targeting 119. A prospective solution is to switch conventional oral administration of metformin to the use of metformin-containing nanomedicines. Nanomedicines could deliver metformin to targeted tissues or cells to realize spatiotemporal immunometabolic regulation at specific sites of interest, meanwhile, nanomedicines can improve bioavailability and pharmacokinetics of metformin. Its optimal doses can be reduced, in this way, its side effects can be minimized. Therefore, the challenges associated with traditional direct administration of metformin could be overcome via the nanomedicines 120. The role of metformin in clinical cancer treatment could be demystified and its effectiveness in eradicating cancer could be enhanced via well-controlled nanomedicines.

## Metformin-based nanomedicines in reprogramming the tumor microenvironment

**Figure 3:**
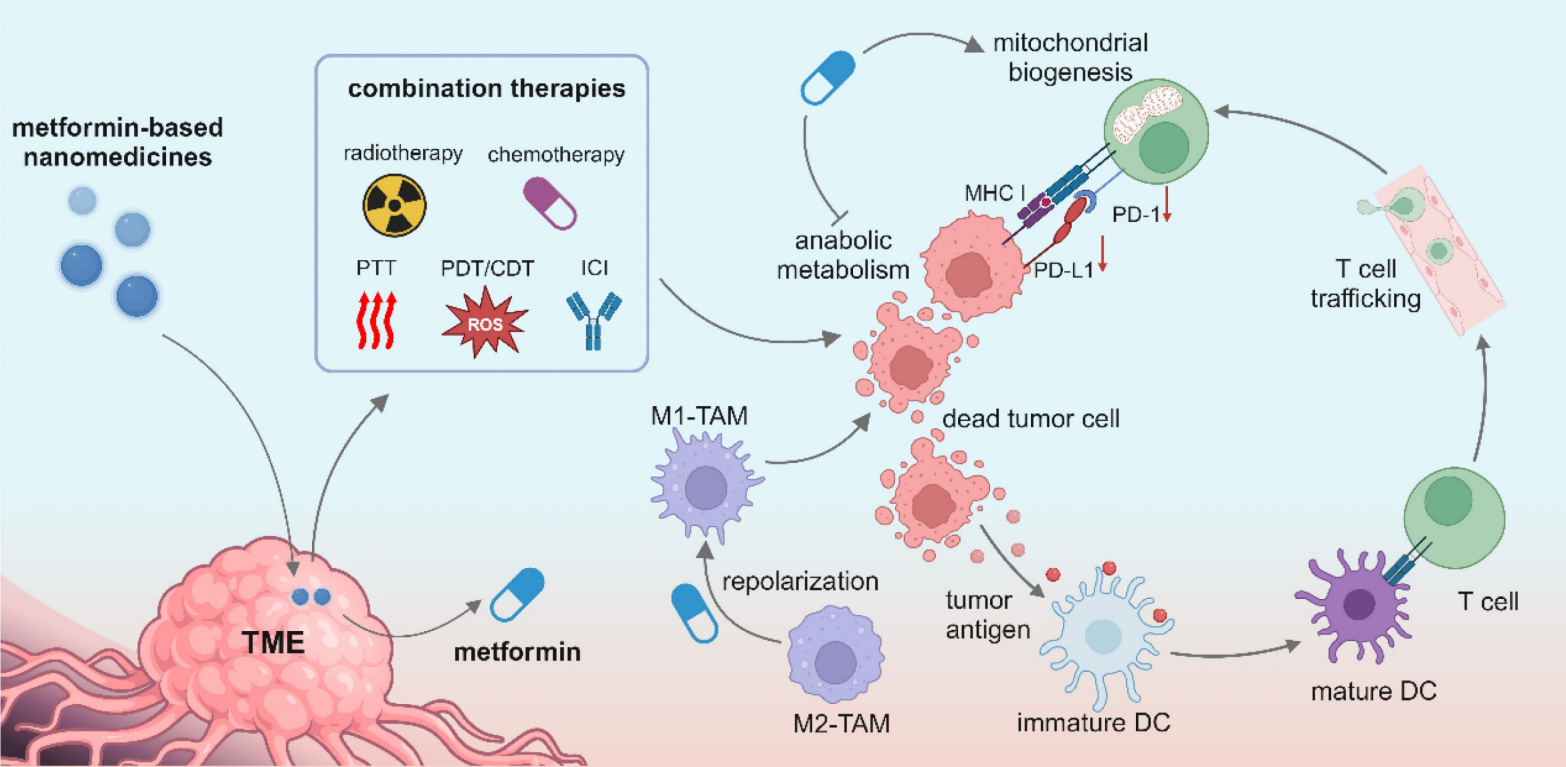
Illustration of metformin-based nanomedicines in combination with other therapeutic modalities for reprogramming the tumor microenvironment. Created in https://BioRender.com.

Nanomedicine is rapidly revolutionizing cancer treatment methods because of its multifunctionality and accommodation of multiple drugs within one nanocarrier, and it opens a new avenue for treating advanced cancers such as triple-negative breast cancer. Great efforts have been made to pursue smart nanomedicines which could preferably accumulate in tumor sites and specifically recognize targeted cells, thereby reducing the damage to normal cells 121. Meanwhile, a variety of combination therapies can be realized in synergy with nanomedicine, including immunotherapy, radiotherapy, chemotherapy, CDT, PDT and PTT (**Figure 3**). Metformin-derived nanomedicines are predominantly developed for targeting tumor cells, and they have been recently used to target immune cells and reprogram the immunosuppressive TME. In this section, we discuss the use of metformin-derived nanomedicines to reprogram a suppressive TME and promote tumor regression (**Table 2**).

### Improving metformin delivery efficiency via nanocarriers

Oral metformin is predominately absorbed by gastrointestinal tracts, and it exhibits unspecific biodistribution due to the broad presence of its corresponding transporters 146. Besides, high hydrophilicity and rapid renal elimination of metformin lead to poor cellular permeation and short half-time in blood 147, 148. All of these above factors restrain the entrance of metformin to cancer cells in which it could interact with mitochondria and activate the AMPK pathway for tumor-suppressive effects. Achieving effective therapeutic outcomes requires enhanced pharmacological properties and sufficient drug accumulation in tumor tissues 149. As a result, there is growing interest in developing nanomedicines that improve pharmacokinetics, targeting, and bioavailability of metformin by encapsulating it in nano-scale carriers 150.

Nanomedicines could prolong half-life and improve tissue distribution of metformin 151. The EPR effect has been the primary mechanism for passive accumulation of nanomedicines in tumor tissues via leaky blood vessels and abnormal lymphatics since 1987 152. A novel concept, active transport and retention (ATR) is proposed. During ATR, tumor endothelial cells can actively transport nanomedicines from the bloodstream to tumor sites through transcytosis 153.

Additional active transfer mechanisms include vesicular transport and blood vessel leakage facilitated by neutrophil extravasation, which help nanomedicines enter tumor tissues 154. However, ATR is not universally accepted for different types of solid tumors, thus personalized nanomedicines have been developed to tailor to specific biological characteristics of different cancer types 155. Active targeting molecules for specific biological characteristics of tumors, such as antibodies, peptides, and aptamers, have been identified and incorporated into nanomedicines to increase their binding affinity to tumor cells, thus enhancing tumor accumulation 156. Similar active targeting strategies could hold promise in targeting immune cells 157, 158. It is important to note that evidence from the past decade supports that only 0.7% of nanoparticles successfully reach tumor tissues due to their elimination via the mononuclear phagocytic system 159. Thus, optimization of the size, charge, and surface coating of nanocarriers is crucial to reduce their uptake by mononuclear phagocytes in the liver and spleen 160. Therefore, nanocarriers with optimized physio-chemical properties could act as an effective delivery system for metformin.

Nanomedicines could also enhance overall biocompatibility and pharmacokinetics of metformin while achieving a high localized concentration at tumor sites, resulting in improved efficacy and reduced toxicity 29, 159. Over the past decades, a variety of advanced nanocarriers have been developed to deliver therapeutics to tumor sites 161-163. Polymeric nanoparticles are nanoscale biomaterials made from polymers, and poly lactic-co-glycolic acid (PLGA) nanoparticles are the most widely used due to their excellent biocompatibility 164-167. PLGA nanoparticles have been reported to protect metformin from early release and realize sustainable release over 160 hours 168. It is noted that polymetformin (Polymet) is a polymeric form of metformin with positive charge. Polymet has been employed to deliver siRNA and form nano-composites with therapeutic photothermal agents for tumor treatment 124, 125. Liposomes are an effective drug delivery system with improved pharmacokinetics and bioavailability 169. It was reported that liposomes could improve the entrapment efficiency of metformin (~65%) 170. Micelles are self-assembled nanoscale spheres composed of amphiphilic molecules, and they have a hydrophobic tail and a hydrophilic head 171. Metformin-containing polymeric micelles could release metformin via pH-responsiveness and exhibit more potent cytotoxicity against breast cancer 172. Hydrogels are highly biocompatible and biodegradable networks of cross-linked polymer chains 173. Beyond controlled and sustained release of metformin, hydrogels have been reported to realize transdermal administration of metformin, which could be a potential administration route for metformin 174. Notably, nanomedicines have been successful in prolonging blood circulation, enhancing tissue accumulation and penetration, facilitating cellular internalization, and ultimately controlling drug release 175, 176. In this context, the dimension, shape, and surface characteristics of nanocarriers could be meticulously fine-tuned to achieve great effectiveness of metformin-based nanomedicines.

### Metformin-loaded nanomedicines inhibit tumor growth in combination with other treatments

**Figure 4:**
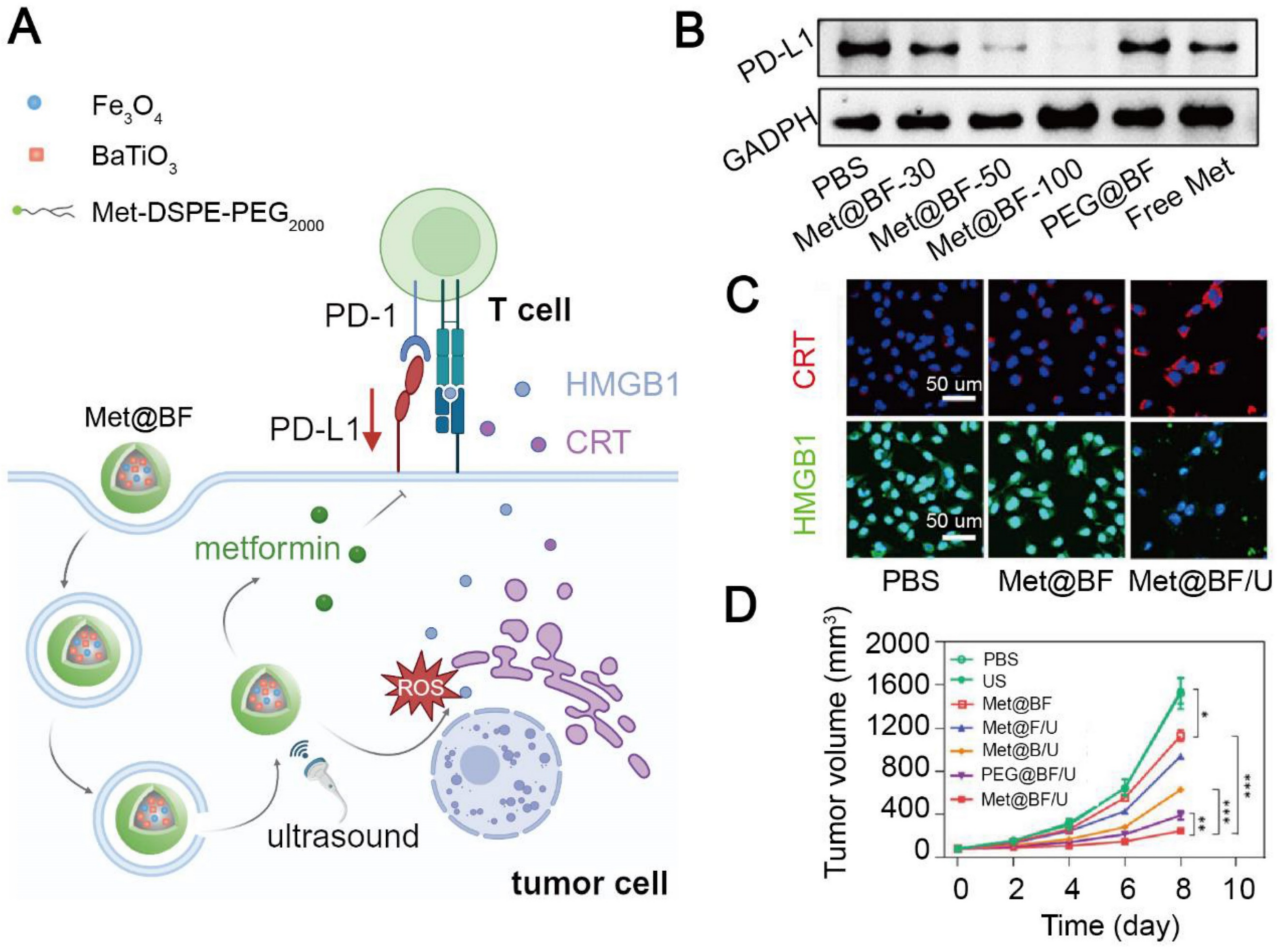
Metformin-based nanomedicine in combination with chemodynamic therapy. (A) Schematic illustration of the mechanism of action of Met@BF nanohybrids for cancer therapy. Created in https://BioRender.com. (B) Western blotting of PD-L1 after different treatments. (C) Confocal fluorescence images of CRT and HMGB1 released from dead tumor cells treated with Met@BF upon ultrasound irradiation. (D) The degree of tumor growth inhibition in B16F10 tumor-bearing mice after different treatments. Adapted with permission from 144, copyright 2024 American Chemical Society.

Blocking the PD-L1/PD-1 engagement can resume proliferation of T cell and recover their function. However, PD-1-based ICIs including pembrolizumab and nivolumab are costly for cancer treatment (over 100,000 dollars per year) 177. In addition, unspecific engagement of anti-PD-1 antibodies to PD-1 expressed on normal cells can lead to systemic toxicity 178. Therefore, cheap and safe agents have been developed to prevent the immunosuppressive signaling from PD-L1/PD-1 interaction. Wang et al. constructed a stimuli-responsive nanohybrid, Met@BF with BaTiO_3_ and Fe_3_O_4_ nanoparticles, to deliver metformin and induce CDT in melanoma 144. The imine bond moiety of Met@BF realized a charge-reversal in an acidic TME: Met@BF exhibited a negative potential to reduce macrophage capture during blood circulation, but a positive potential in an acidic tumor site to facilitate tumor cell endocytosis. In addition, BaTiO_3_ produced H_2_O_2_ upon ultrasonic irradiation to increase Fe_3_O_4_-mediated ROS generation, inducing an ICD effect. Dead tumor cells released HMGB1 and CRT to promote dendritic cell maturation and subsequent T cell activation, triggering anti-tumor immunity. Finally, released metformin from the Met@BF nanohybrid at tumor sites significantly downregulated the PD-L1 level on tumor cells and subsequently enhanced T cell infiltration, thus curbing the development of primary and metastatic B16F10 tumor masses in mice (**Figure 4**). However, nanomedicines without tumor targetability may induce off-target effects and systemic toxicity. Hu et al. constructed tumor cell-targeting nanoparticles, MA-pepA-Ce6 NPs, to specifically deliver metformin and chlorin e6 (Ce6) as a photosensitizer to breast cancer cells. The nanoparticles were cleaved by abundant matrix metalloproteinase-2 within the TME to release peptide-conjugated metformin and integrin α_v_β_3_ ligand-modified Ce6. Ce6 was bound to α_v_β_3_-overexpressed tumor cells and induce an ICD effect under radiation. Metformin with a positive potential was easily internalized by tumor cells to promote PD-L1 proteasomal degradation and increase IFN-γ secretion from CD8^+^ T cells. Therefore, MA-pepA-Ce6 NPs mitigated the immunosuppressive level in the TME via downregulating PD-L1 and inducing ICD and inhibited 4T1 tumor growth without exerting significant systemic toxicity 122.

Apart from ameliorating an immunosuppressive TME via promoting PD-L1 degradation, metformin exhibits direct cytotoxic effects on tumor cells. Jafari et al. constructed folate-modified-PLGA-polyethylene glycol (PEG) nanoparticles to improve blood circulation and bioavailability of metformin 128. The nano-formulated metformin induced more pronounced apoptosis of breast cancer cells than free metformin. However, metformin-dependent cytotoxicity is not sufficient to induce tumor regression. Therefore, combining metformin-based nanomedicines with other treatment modalities including radiotherapy and chemotherapy could lead to more potent ICD.

**Figure 5:**
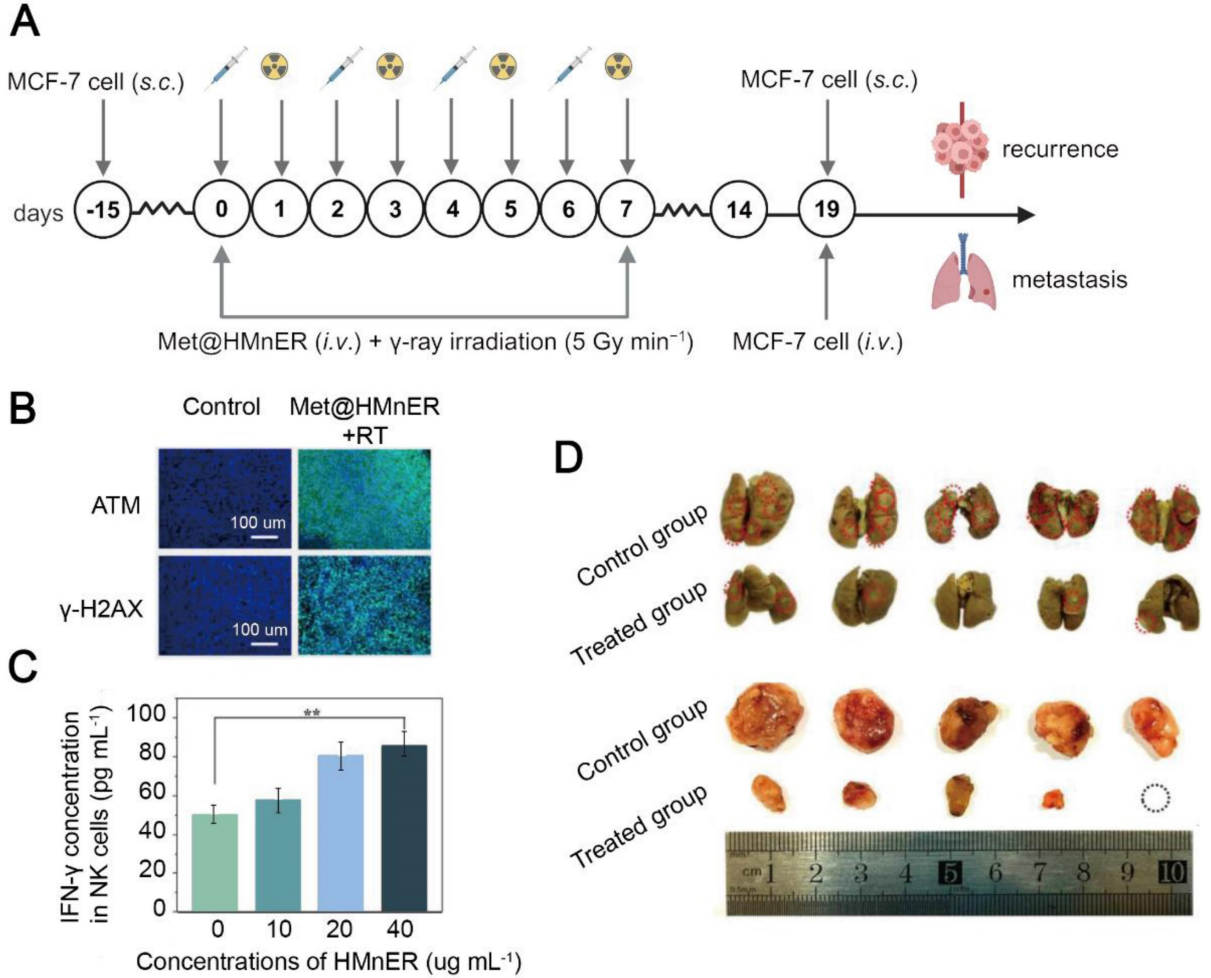
Metformin-based nanomedicine in combination with radiotherapy. (A) Schematic illustration of Met@HMnER to inhibit cancer recurrence and metastasis in the MCF7 tumor-bearing mice. Created in https://BioRender.com. (B) Fluorescence microscope images for ATM and γ-H2AX. Elevation in the expression of both of them indicated significant DNA damage and enhanced radiotherapy sensitization after treatment with Met@HMnER. (C) The expression levels of IFN-γ secreted by NK cells through activating the cGAS-STING pathway by Met@HMnER. (D) Photos of lungs (above) and recurrent tumors (below) after treatment with Met@HMnER, confirming its anti-recurrence and anti-metastasis efficacy. Adapted with permission from 138, copyright 2023 Elsevier Ltd.

Hypoxia is one of the culprits for a low efficacy of clinical radiotherapy 179, and insufficient oxygen in the severely hypoxic solid tumor microenvironment results in a lower level of ROS generated from MnO_2_, a traditional radiosensitizer 180. It was found that metformin sensitized radiotherapy by blocking mitochondrial complex I, thereby decreasing the oxygen consumption in cancer cells 62. To improve the efficiency in the delivery of metformin to tumor tissues, Yang et al. employed natural extracellular vesicles as nanocarriers to realize prolonged circulation and immune escape to increase cellular uptake of the metformin-containing nanomedicines 181. MnO_2_ nanocomposites were first constructed from hollow MnO_2_ nanoparticles. Metformin and the MnO_2_ nanocomposites were encapsulated by RGD-modified extracellular vesicles to form a radiosensitive nanomedicine, Met@HMnER 138. Tumor cell uptake of Met@HMnER was significantly enhanced through RGD-α_v_β_3_ binding, and oxygen and Mn^2+^ were released via stimuli-responsiveness from excessive H_2_O_2_ and glutathione (GSH) within the TME, respectively. Promotion of oxygen generation in conjunction with metformin-mediated inhibition of oxygen consumption alleviated hypoxia in tumor tissues and enhanced the radiotherapeutic efficacy, while Mn^2+^ promoted IFN-γ secretion from NK cells via activating the cyclic guanosine monophosphate-adenosine monophosphate synthase (cGAS)-stimulator of interferon genes (STING) pathway 182. Met@HMnER simultaneously augmented innate anti-tumor immunity and improved the radiotherapy efficacy to inhibit tumor metastasis and recurrence in MCF-7 tumor-bearing mice (**Figure 5**). Notably, metformin has also been reported to activate the cGAS-STING pathway through AXIN1-dependent STING stabilization. Dou et al. constructed Mn-MSN@Met-M nanoparticles with a coating of cancer cell membranes to specifically deliver metformin and Mn^2+^ to lung cancer cells in the mice with LKB1 mutation. The loss of LKB1 in the mice usually leads to inhibition of the STING pathway and reduction in IFN-β secretion 183. Intravenous administration of the nanomedicine resulted in an increase in localized enrichment of metformin and Mn^2+^ in cancer cells, both of which synergistically upregulated the cGAS-STING pathway and enhanced T cell tumor-killing ability 145.

Metformin has also been combined with chemotherapeutic agents. Cisplatin is one of the most popular agents for chemotherapy, but its efficacy is diminished due to upregulation of DNA repair in tumor cells. Nucleotide excision repair and interstrand crosslink repair are credited to the excision repair cross-complementing 1 (ERCC1) protein 184. To reduce chemoresistance of cisplatin in lung cancer, Yang et al. constructed self-assembled nanoparticles to deliver cisplatin and metformin in a nanomedicine format, HA-CDDP/PMet. This nanomedicine specifically targeted cancer cells through hyaluronic acid-CD44 binding. Remarkably, metformin inhibited the mTOR activity and helped downregulating the expression of ERCC1, thus inhibiting DNA repair and overcoming the resistance of cisplatin-based chemotherapy in the LLC tumor-bearing mice 123. However, DNA damage can induce an elevation in the intracellular Ca^2+^ level and activation of AMPK for DNA repair via inhibiting exonuclease 1 (EXO1) and p53-binding protein 1 (53BP1) 185, 186. Arguably, metformin may assist in DNA repair rather than promote cisplatin-mediated DNA damage, and the precise mechanism of action of metformin-enhanced cisplatin chemotherapy needs to be unveiled. In another study on the combination of metformin with cisplatin by Saber et al., metabolic changes in tumor cells were detected. Self-assembled nano-cubosomes were prepared to encapsulate metformin and cisplatin to treat colorectal cancer 187. Metformin was released from the nano-cubosomes to inhibit glycolysis and ATP production via downregulating the mTOR activity, thus eliciting oxidative stress and apoptosis and ultimately enhancing cytotoxicity of cisplatin. Metformin-mediated mTOR inhibition suppressed tumorigenesis-associated glycolysis and promoted tumor cell apoptosis, confirming a critical role of metformin in combinational cancer treatments.

Metformin as an adjuvant exhibits an anti-tumor effect, while its targetability and bioavailability are often quite poor after direct administration. Metformin-containing nanomedicines can selectively deliver metformin to tumor cells to downregulate PD-L1 and inhibit mitochondrial respiration. Notably, metformin alone could not exert potent cytotoxic effects on tumor cells, while it could be combined with ICD-inducing treatment modalities such as PDT, radiotherapy and chemotherapy. Continuous efforts should be concentrated on revealing the mechanisms of reprogramming an immunosuppressive TME via metformin in nanomedicines.

### Metformin-loaded nanomedicines promote T cell oxidative metabolism and memory differentiation

**Figure 6:**
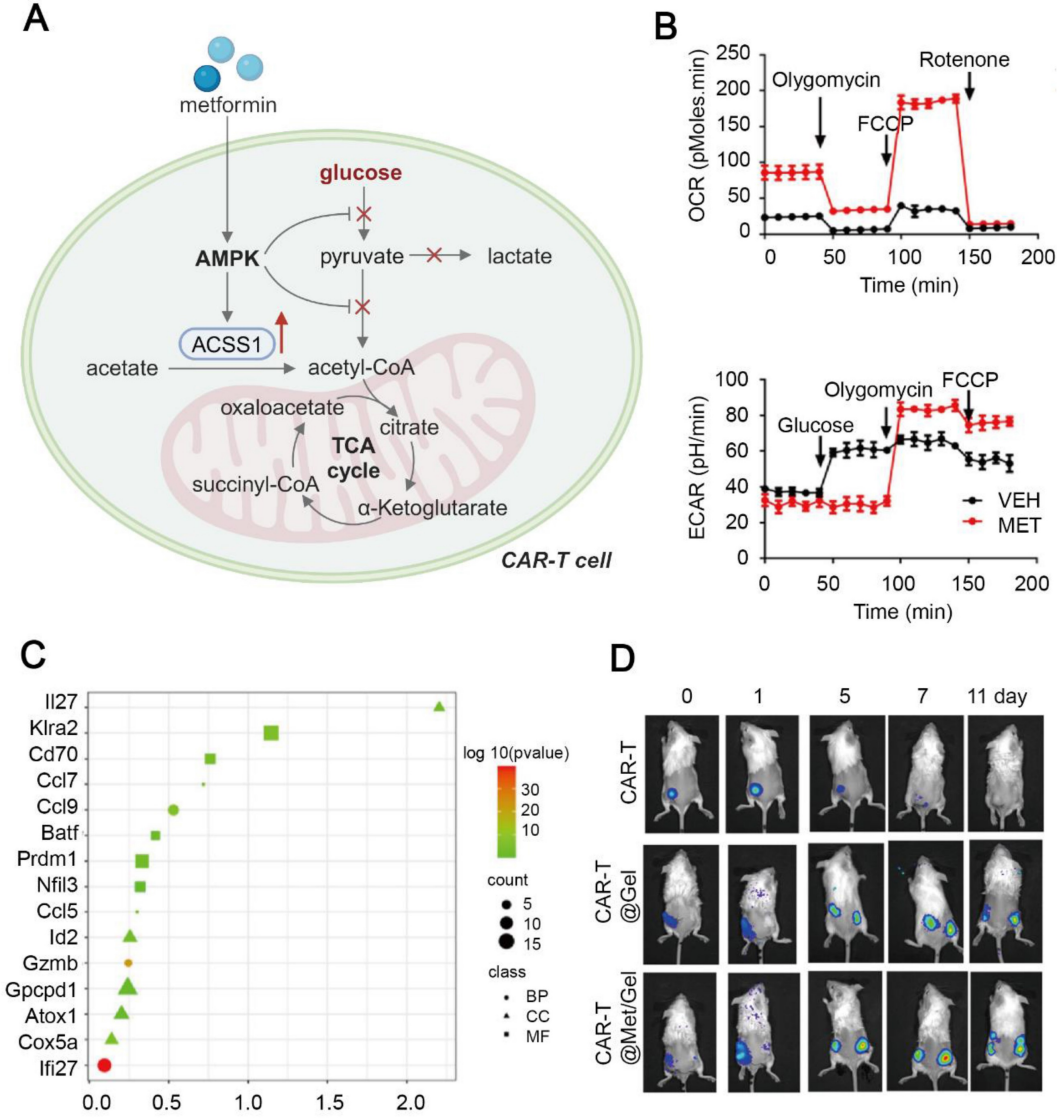
Metformin-based hydrogel scaffold to promote anti-tumor immune response of CAR-T cells. (A) Schematic illustration of metabolic reprogramming in CAR-T cells treated with the metformin-containing hydrogel. Created in https://BioRender.com. (B) Metabolic flux analysis of the oxygen consumption rate and the extracellular acidification rate in CAR-T cells treated with metformin, indicating suppression of glycolysis and enhancement in oxidative phosphorylation. (C) Upregulation of memory phenotype-related transcripts in CAR-T cells treated with CAR-T@Met/gel. (D) Bioluminescence images of CAR-T cell proliferation in primary and distant lesions in the HGC-27 tumor-bearing mice. Adapted with permission from 67, copyright 2023 Elsevier Ltd.

Immunometabolic modulation of T cells via metformin has been extensively explored. Polymeric metformin has been used to enhance T cell infiltration via AMPK activation and improve the TME, and it has been combined with ICI therapy in colorectal cancer treatment 129. Tumor relapse is a predominant indicator of poor outcomes in cancer patients, while long-living T_m_ cells can prevent tumors from recurrence. It is well established that maintenance of T_m_ cells is principally dependent on mitochondrial metabolism, such as OXPHOS and FAO 188. Therefore, Chao et al. constructed a hydrogel scaffold to store and gradually release chimeric antigen receptor T (CAR-T) cells and metformin, and implanted the scaffold in the post-resection tumor site to evaluate the anti-tumor effect 67. Interestingly, released metformin from the scaffold inhibited glycolysis and OXPHOS in tumor cells, while it helped strengthening the TCA cycle and mitochondrial respiration in the T cells. In CAR-T cells, upregulation of ACSS1 boosted acetyl-CoA production, fueling the TCA cycle and OXPHOS. Metabolic reprogramming of CAR-T cells enhanced their proliferation and secretion of effector cytokines. The scaffold fine-tuned CAR-T cells into a more persistent and memory-like phenotype, resulting in significant inhibition of primary and metastatic tumors in the HGC-27 tumor-bearing mice (**Figure 6**). However, distinct metabolic influences by metformin on cancer cells and T cells remain unknown. We believe metformin improves T cell viability via altering their engagement with cancer cells, since metformin fails to exert such effects in the absence of cancer cells. Interestingly, different concentrations of metformin can exert absolutely different effects on the mitochondrial activity 189, indicating the importance of designing a proper dose of metformin to tumor cells and T cells for activating effective anti-tumor effects.

The exposure of tumor antigens to T cells is required for T cell activation, but long-term stimulation by tumor antigens as anti-tumor vaccines can exhaust T cells and block their differentiation to a memory-like phenotype. Metformin can downregulate the expression level of immunosuppressive PD-1 and prolong T_m_ cell survival by strengthening their mitochondrial function and elevating their FAO level. Luo et al. developed a metformin-based tumor vaccine to induce generation of T_m_ cells 126. They initially treated the tumor lesion with PTT to acquire anti-tumor immunogenicity. The tumor antigens were collected and co-encapsulated with metformin and hollow gold nanoparticles as a photothermal agent into biodegradable PLGA microspheres to obtain TA-Met@MS as a vaccine. TA-Met@MS released pulsed tumor antigens and metformin under near infrared radiation to promote T cell activation and subsequent central T_m_ cell formation via interfering with FAO. Thus, TA-Met@MS pronouncedly inhibited tumor growth and metastasis and a great number of CD8^+^ T_m_ cells were produced in the 4T1 and B16F10 tumor-bearing mice. Since effectiveness of cancer vaccines relies on rapid response from T_m_ cells, central T_m_ cells could be endowed with a hyper proliferative ability in response to tumor antigen reencounter 190. Notably, the metformin-activated AMPK signaling pathway promotes catabolism and T_m_ cell differentiation, but it could impair T_eff_ cell differentiation 46, therefore, it is suggested to induce T_m_ cell differentiation via metformin during the T cell contraction phase, which could maximize in maintaining anti-tumor immune response.

Novel T cell-targeting nanomedicines have emerged for cancer treatment. For example, engineered T cells anchored with a nanomedicine resolved the issue of vasculature extravasation of nanomedicines and they precisely acted on T cells without any physiological changes 191. More recently, a tri-specific nano-antibody has been reported to simultaneously bind to tumor cells via targeting PD-L1, and T cells and NK cells via targeting 4-1BB and natural killer group 2 member A (NKG2A) 192. The tri-specific nano-antibody exhibited more effective targetability and better anti-tumor immunity compared with clinical monoclonal antibodies and bispecific monoclonal antibodies. Metformin could be combined with these innovative and effective strategies in the nano-medicinal format for advanced solid tumors.

Emerging immunological evidence indicates precursor exhausted T cells in lymph nodes are emerging as novel targeting candidates, since these precursor cells can respond to ICIs and then proliferate and replenish functional T cells at the tumor site 193. However, upon prolonged stimulation by tumor antigens, precursor exhausted T cells inevitably generate terminally exhausted progeny 194. Mitochondrial dysfunction has been found to be linked to T cell exhaustion 195, 196, and studies have confirmed that PGC-1α-overexpressed T cells exhibit significant improvements in mitochondrial biogenesis and expansion 197. Since metformin can activate PGC-1α via the AMPK signaling pathway, it could be employed to enhance the mitochondrial fitness of precursor exhausted T cells to mitigate the exhaustion process and maintain their tumor-killing ability. However, metformin-containing nanomedicines should be designed and constructed to break through barriers in lymph nodes 198, 199.

Given that T cells are pivotal in driving tumor regression, it is critical to promote their growth, sustain their survival, and boost their production of effector cytokines. With the help of a nanocarrier, metformin as a safe and accessible immunologic adjuvant could be harnessed to improve oxidative metabolism of T cells via activating the AMPK pathway, which can upregulate the associated enzymes and promote mitochondrial biogenesis, thus reinvigorating their anti-tumor immunity and modulating the immunosuppressive TME.

### Metformin-loaded nanomedicines repolarize M2-like tumor-associated macrophages to M1 phenotype

**Figure 7:**
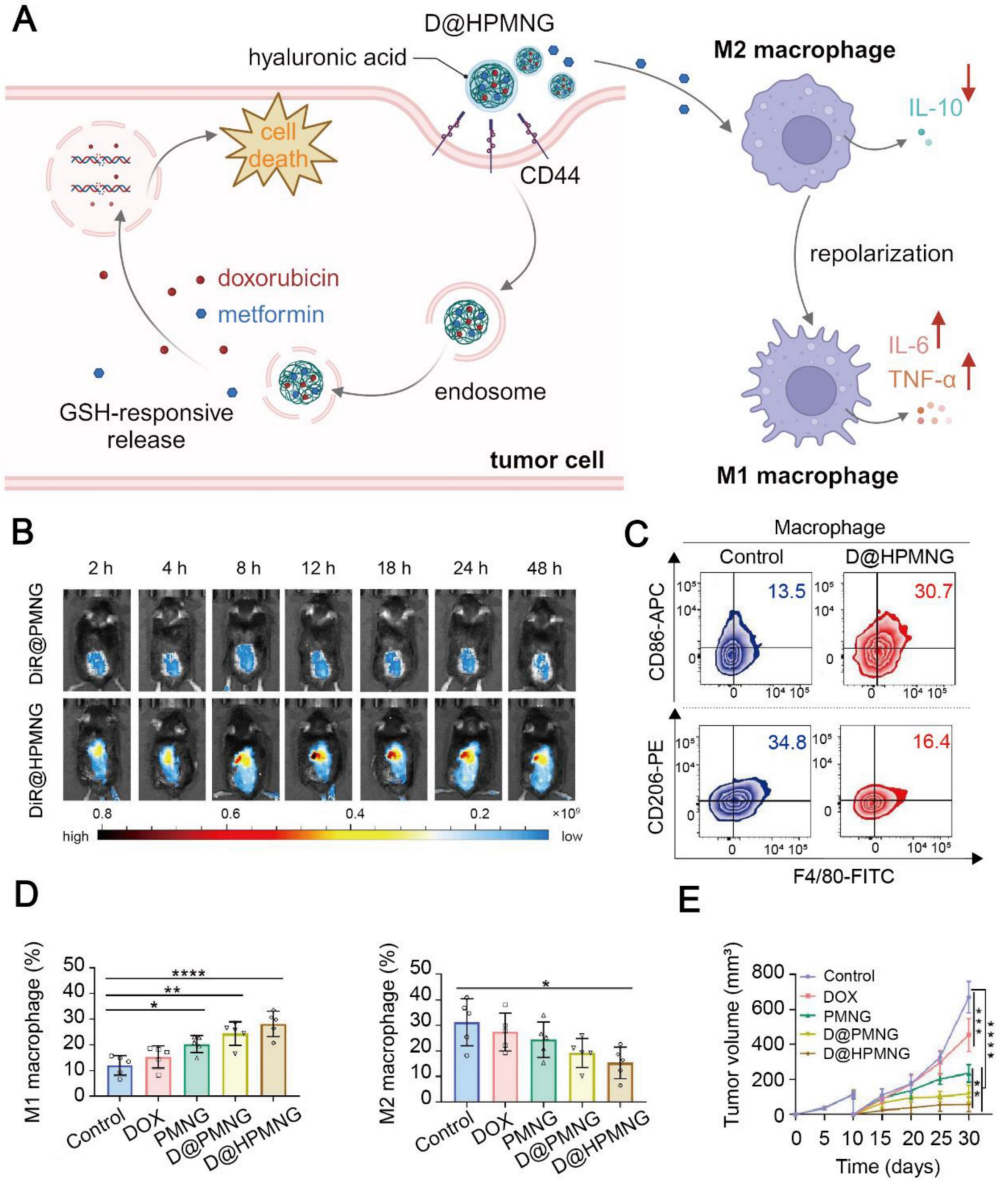
Metformin-based nanomedicine in combination with chemotherapy. (A) Schematic illustration of the mechanism of action for D@HPMNG to combat cancer and repolarize the TAM phenotype. Created in https://BioRender.com. (B) Fluorescence images of biodistribution of DiR-labeled HPMNG nanogels in the mice, indicating excellent targeting ability of the nanogel. Representative flow cytometry plots of M1-like and M2-like TAMs (C) and their quantitative results (D) at recurrent tumor sites after treatment with D@HPMNG and its controls. (E) The recurrent tumor growth profile after different treatments. Adapted with permission from 135, copyright 2024 American Chemical Society.

TAMs are a well-recognized cell population in the TME, and they are usually educated to an M2 phenotype to express PD-L1 and secrete suppressive cytokines, thus inhibiting T cells. The use of metformin via nanomedicines to regulate the TAM phenotype is a promising approach to revitalizing the immunosuppressive TME. Tang et al. constructed a GSH-responsive nanogel, PMNG, from carboxymethyl chitosan, metformin and cystamine 135. The nanogel exhibited prominent deformability, facilitating its penetration into deep tumor tissues 200. PMNG was then loaded with doxorubicin (DOX) and coated with hyaluronic acid (HA) to obtain a D@HPMNG nanomedicine. Tumor-targeting by the nanomedicine was realized via specific interaction between CD44 and HA. The HA coating also prevented D@HPMNG from immune clearance and improved biocompatibility. After the nanomedicine was effectively uptaken by tumor cells, overexpressed GSH in tumor cells immediately cleaved the disulfide bond in D@HPMNG to release DOX to induce tumor cell apoptosis without inducing cardiotoxicity. Besides, D@HPMNG reprogrammed the TME via inducing the M2-to-M1 repolarization of TAMs, increasing the portion of T_eff_ cells, and reducing collagen deposition, ultimately inhibiting tumor growth and relapse in the B16F10 tumor-bearing mice (**Figure 7**). Another metformin-containing nanogel developed by Tian et al. repolarized the TAM phenotype *in vitro* and activation of the AMPK signaling pathway was found to contribute to the phenotype conversion 136.

**Figure 8:**
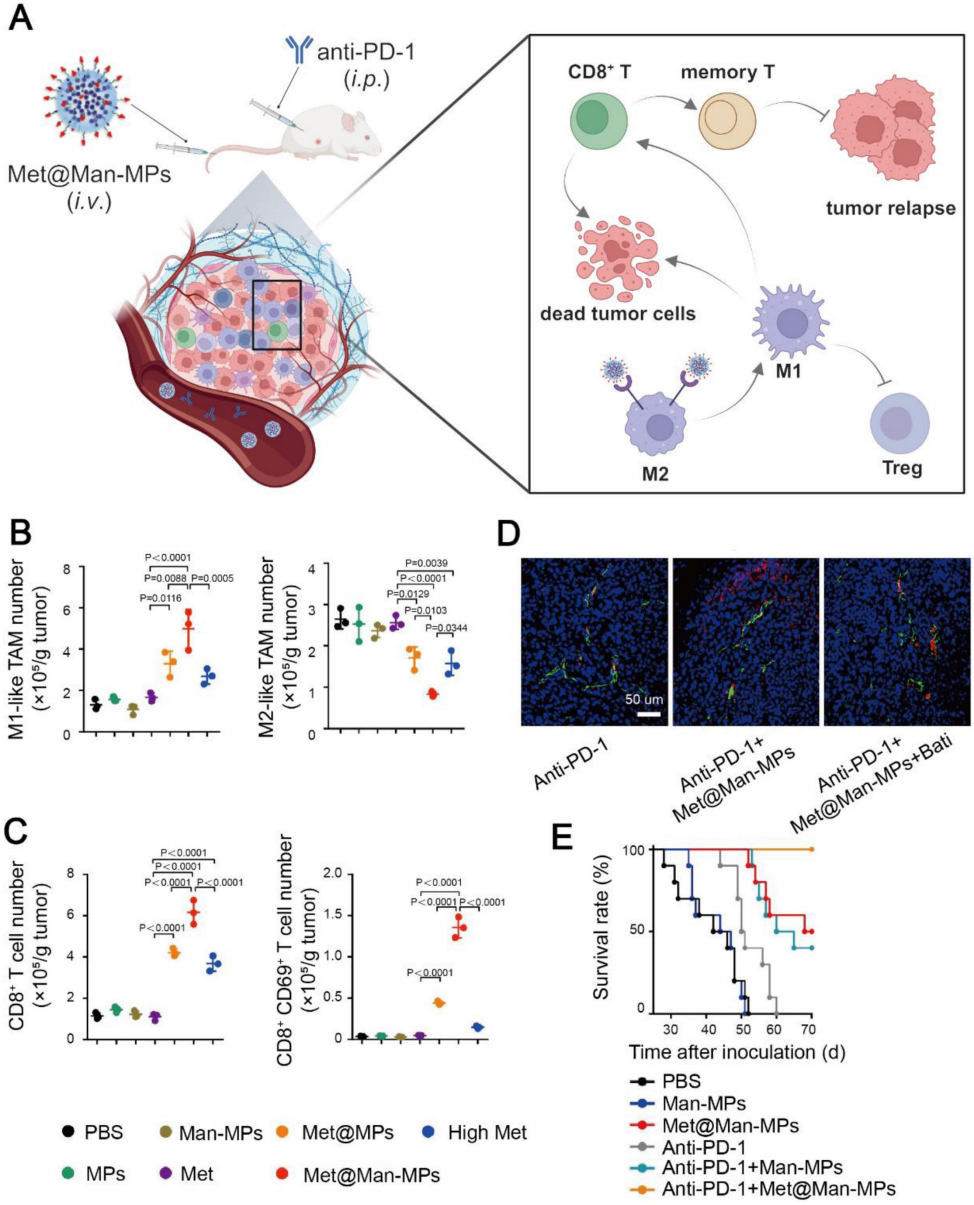
Metformin-based nanomedicine in combination with immunotherapy. (A) Schematic illustration of the anti-tumor mechanism of Met@Man-MPs in synergy with anti-PD-1 antibody. Created in https://BioRender.com. The portion of M1-like TAMs, M2-like TAMs (B) and CD8^+^ T cells (C) in tumor sites after different treatments. (D) Colocalization of the anti-PD-1 antibody (red) and endothelial cells (green), indicating Met@Man-MPs strengthened the penetration of the anti-PD-1 antibody in tumor sites. (E) Survival curves of H22 tumor-bearing mice after different combined treatment methods. Adapted with permission from 140, copyright 2021 Springer Nature.

However, the above nanogels were not specifically delivered to M2-like TAMs. To exclusively study the influence of metformin on TAM repolarization, Wei et al. developed macrophage-derived microparticles to load metformin, and then modified the microparticles with mannose, resulting in a Met@Man-MP nanomedicine. The mannose moiety on the microparticle could specifically bind to overexpressed CD206 on M2-like TAMs 140. Met@Man-MP displayed excellent stability, biocompatibility and safety during blood circulation, and it exhibited a matrix metalloproteinase-like activity to degrade the ECM in the TME. Therefore, Met@Man-MP selectively targeted M2-like TAMs and reshaped them to an M1 phenotype, and it boosted the infiltration of T_eff_ cells as well as anti-PD-1 antibodies through promoting collagen degradation. Interestingly, the decomposition of collagen in the ECM mediated by Met@Man-MP did not result in tumor metastasis. Eventually, Met@Man-MP reprogramed the immunosuppressive TME via repolarizing M2-like TAMs to an M1 phenotype and elevating the portion of T_eff_ cells, and enhanced the effectiveness of ICIs in the H22 tumor-bearing mice (**Figure 8**). Both nanogels and microparticles could repolarize TAMs from an M2 to M1 phenotype, but the specific mechanism of AMPK activation for repolarizing the TAM phenotype via metformin remained to be unveiled.

Experimental results have confirmed that metformin could reshape the TAM phenotype, but more studies should be conducted to verify its effectiveness. The mechanism of conversion of the TAM phenotype is not revealed. Metformin may be directly involved in TAM phenotype repolarization via the AMPK pathway. Alternatively, it may impair the reeducating ability of tumor cells to induce M2-like TAM differentiation by blocking the secretion of immunosuppressive cytokines. A comprehensive understanding of the immunometabolism-modulating mechanism of metformin is conducive to developing potent metformin-containing nanomedicines in reprogramming the TME.

## Conclusion

Metformin, a first-line treatment drug for T2DM to lower the blood glucose concentration through AMPK activation, has recently gained attention as a potential adjuvant in cancer therapy due to its direct and indirect anti-tumor effects. Metformin-mediated AMPK activation can induce PD-L1 degradation and interfere with anabolic processes in tumor cells via inhibiting mTOR. Additionally, inhibition of mitochondrial complex I by metformin can reduce oxygen consumption in tumor cells and remodel a hypoxic TME. Inspiringly, metformin reprograms the TME via enhancing the effector activity of anti-tumor immune cells. For example, AMPK activation in T cells favors energy metabolism and promotes memory phenotype formation, and it also contributes to anti-tumor repolarization of TAMs. However, direct administration of metformin has shown limited benefits in cancer therapy, which may be due to significant variations in the local concentration of metformin at tumor sites. To address this issue, metformin-containing nanomedicines have been developed to increase its localized concentration in tumor tissues, thereby revealing its role as an anti-tumor adjuvant and confirming its tumor-suppressing effects. The introduction of the nanomedicine formulation endows metformin with improved targetability and bioavailability through diversified strategies for targeting and responsive release in response to characteristic stimuli in the TME, in this way, the toxicity of metformin can be significantly diminished. A variety of nanomaterials have been explored to encapsulate metformin in nanomedicines. More encouragingly, metformin combined with other therapeutic drugs can be incorporated into one single nanomedicine to realize combination therapy. In summary, repurposing metformin in a nano-medicinal formulation holds great potential for effectively reprogramming an immunosuppressive TME and comprehensively activating anti-tumor immunity.

**Figure 9:**
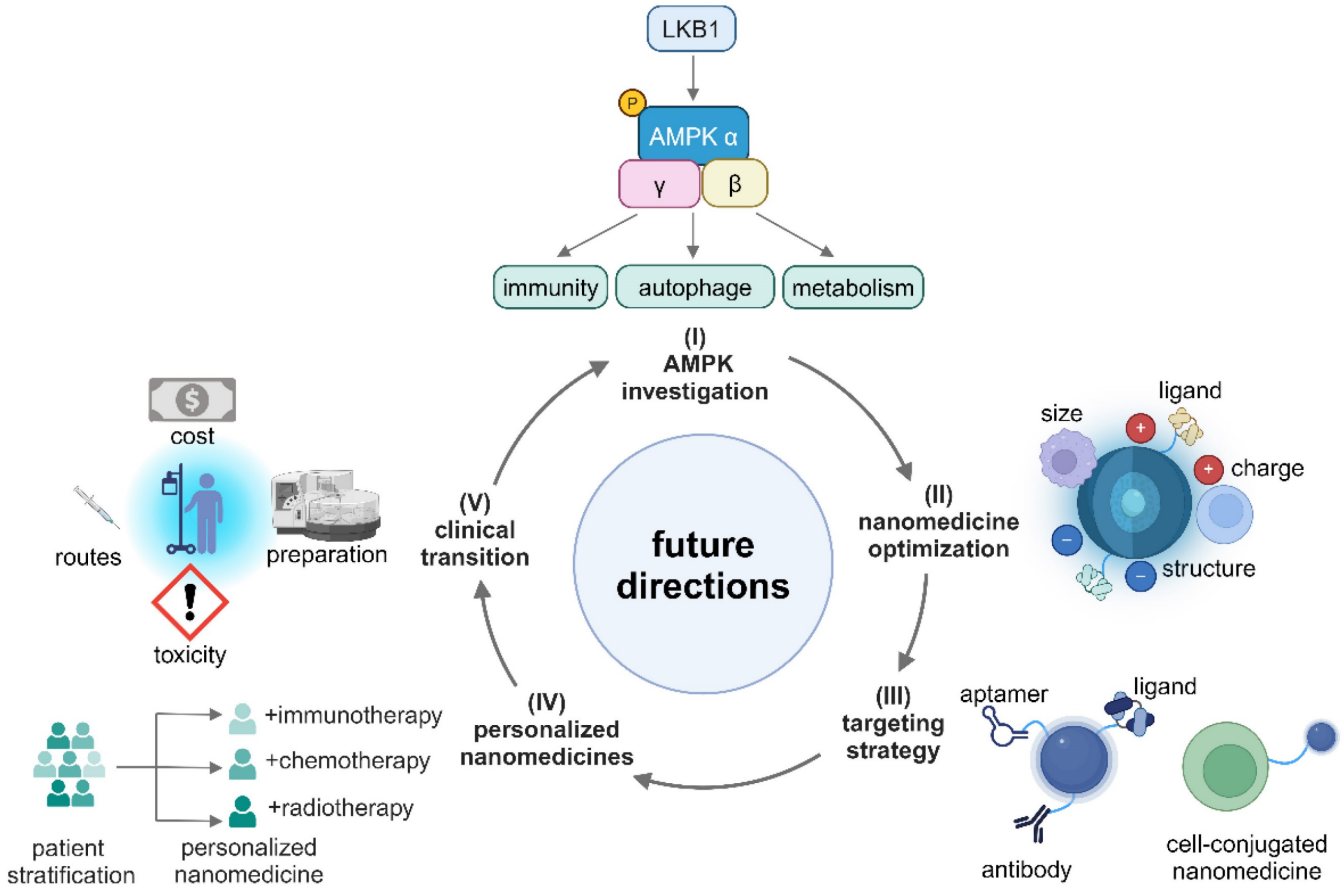
Illustration of the future directions of metformin-based nanomedicines. Created in https://BioRender.com.

Although metformin-containing nanomedicines have shown promise in reprogramming an immunosuppressive TME, there are very few fundamental mechanistic studies in this area. To fully leverage the potential of metformin in cancer nanomedicines, the following future research directions are highly recommended (**Figure 9**). (I) Conducting systematical studies on the AMPK signaling network to identify specific targets that induce tumor-suppressive effects in different immune cells; (II) Enhancing efficiencies in accumulation and penetration of nanomedicines in the TME by optimizing their size, charge, coating, and ligands. In addition, exploring and designing immune-regulating nanomaterials for metformin-containing nanomedicines is conducive to enhancing anti-tumor immune response; (III) Improving active targetability and reducing off-target effects of metformin-based nanomedicines via exploring high-affinity peptides, antibodies, aptamers and developing cell-conjugated nanomedicines; (IV) Developing personalized nanomedicines through patient stratification by tissue biomarkers and biological characteristics of different cancer types; (V) Finally, reducing the cost, simplifying the preparation process, selecting the best administration routes, and mitigating toxicities from nano-formulations so that the nanomedicines can be readily translated into clinical use.

In conclusion, metformin in a nanomedicine formulation has emerged as a promising adjuvant to activate the AMPK signaling pathway which is a key regulator in cellular metabolic activities, energy production and immune response, and it can reprogram the TME by remodeling an immunologically cold TME into an immunologically hot one to reactivate the suppressed anti-tumor immunity. In the nanomedicine formulation, metformin can work in synergy with other therapeutic agents or modalities to eliminate malignancies. The mechanisms of regulating immunometabolism within the TME via AMPK activation by metformin remain mysterious because of the complex interplay between the components of the AMPK signaling pathway and unveiling of these mechanisms could offer great potential in restoring suppressed anti-tumor immunity within the TME. Overall, repurposing metformin in a nanomedicine formulation to reprogram the TME will advance cancer treatment and benefit cancer patients.

## Figures and Tables

**Table 1:** Metformin as an adjuvant cancer therapy in clinical trials

Stage	Treatment	Indication	NCT code
Early phase I	Atorvastatin + Metformin	Operable breast cancer	NCT01980823
Phase I	Erlotinib + Metformin	Triple negative breast cancer	NCT01650506
Phase I	Sapanisertib + Metformin	Advanced solid tumors	NCT03017833 108
Phase I	Temsirolimus + Metformin	Advanced solid tumors	NCT00659568
Phase II	Levonorgestrel-releasing intrauterine device + Metformin	Endometrial cancer	NCT02035787
Phase II	Capecitabine + Radiotherapy + Metformin	Locally advanced rectal cancer	NCT02437656
Phase II	Abiraterone + Metformin	Metastatic prostate cancer	NCT01677897 109
Phase II	Letrozole + Metformin	ER (+) postmenopausal breast cancer	NCT01589367 110
Phase II	Docetaxel + Metformin	Castration-resistant prostate cancer	NCT01796028 111
Phase II	Letrozole + Metformin	Postmenopausal breast cancer	NCT05053841 112
Phase II	Standard chemotherapy + Metformin	Metastatic breast cancer.	NCT01310231 113
Phase II	Nivolumab + Metformin	Metastatic colon cancer	NCT03800602 43
Phase III	Leucovorin + Fluorouracil + Oxaliplatin + Metformin	Metastatic colon cancer	NCT05921942

**Table 2:** Metformin-based nanomedicines reprogram the tumor microenvironment

Material type	Nanoformulation	Function	Combination therapy	Indication	Refs.
**Peptide-based nanoparticle**	MA-pepA-Ce6	MMP-2 responsiveness; α_v_β_3_-mediated tumor targeting; downregulation of PD-L1 expression	PDT	Breast cancer	122
**Polymeric Nanoparticle**	HA-CDDP/PMet	Hyaluronic acid-mediated tumor targeting; reversal of chemotherapy resistance; inducing tumor cell apoptosis	Chemotherapy	Lung cancer	123
	aPD-L1-PolyMet/BPN	Anti-PD-L1 antibody-mediated tumor targeting; inhibiting primary and abscopal tumor growth and metastasis	PTT; chemotherapy and immunotherapy	Breast cancer	124
	LPH-PolyMet-siVEGF	Inducing tumor cell autophagy and apoptosis through downregulating mTOR; decreasing VEGF production	Gene therapy	Non-small-cell lung cancer	125
	TA-Met@MS	Pulsed release of metformin and tumor antigens; promoting FAO and memory phenotype differentiation of CD8^+^ T cells	PTT	Breast cancer and melanoma	126
	Metformin-loaded PLGA-PEG NPs	Inhibition of mTOR and reduction in telomerase reverse transcriptase expression; G1 phase arrest; inducing apoptosis	--	Breast cancer	127
	Met-loaded FA-PLGA-PEG NPs	Folate-mediated tumor targeting; upregulating the expression of pro-apoptotic Bax, Caspase 7, Caspase 3, p53 and anti-apoptotic Bcl-2	--	Breast cancer	128
	Polymet	Sensitization of anti-PD-L1 antibody; increasing IFN-γ production and infiltration of T cells; increasing anti-tumor intestinal microbes	--	Colorectal cancer	129
**Micelle**	FucOMDs	Fucoidan-mediated premetastatic site targeting; inhibition of adhesion of CTCs to endothelial cells; downregulating the expression of fibronectin and MMP-9	Chemotherapy	Breast cancer	130
	PMD	Downregulating PD-L1 expression; inhibiting T_reg_ cell suppressive activity; promoting DC maturation	Chemotherapy; Immunotherapy	Breast cancer	131
**Liposome**	IR775@Met@Lip	Reversal of hypoxia and enhancing PDT; downregulating PD-L1 expression; enhancing IFN-γ secretion and infiltration of CD8^+^ T cells	PDT	Colorectal cancer	132
	Met-HCe6-Liposome	Sustainable release of metformin in tumor cells; improving hypoxia	PDT	Colorectal cancer	133
	Met-oxa (IV)-liposome	Relieving hypoxia; repolarizing TAMs to an M1 phenotype	Chemotherapy; Immunotherapy	Colorectal cancer	134
**Hydrogel**	D@HPMNG	GSH responsiveness; hyaluronic acid-mediated tumor targeting; repolarization of TAMs to an M1 phenotype; enhancing DC maturation and CD8^+^ T cell infiltration	Chemotherapy	Melanoma	135
	CAR-T@Met/gel	Sustain release of CAR-T cells and metformin; inhibiting glycolysis and OXPHOS in tumor cells; upregulating ACSS1 expression and promotion of OXPHOS and memory phenotype differentiation in CAR-T cells	Immunotherapy	Gastric carcinoma	67
	PMI	Reshaping an M2-like TAM phenotype; downregulating PD-L1 expression in tumor cells; enhancing the infiltration of CD8^+^ T cells	Immunotherapy	Breast cancer	136
	MCGPD/RGD NPs	MMP-2 responsiveness; RGD peptide-mediated tumor cell targeting; improving the hypoxia; reducing ATP production	PDT; PTT and chemotherapy	Breast cancer	137
**Extracellular vesicles**	Met@HMnER	Oxygen generation and hypoxia alleviation; sensitizing radiotherapy; G2/M phase arrest; activating the cGAS-STING pathway	Radiotherapy	Breast cancer	138
	SPI@hEL-RS17 NPs	RS17 peptide-mediated tumor targeting; inhibiting pyruvate kinase-M2 and damaging mitochondria; enhancing macrophage phagocytosis	PDT and chemotherapy	Breast cancer and melanoma	139
	Met@Man-MP	Mannose-mediated M2-like TAM targeting; repolarization of TAMs to an M1 phenotype; recruitment of CD8^+^ T cells; reducing immunosuppressive MDSCs and T_reg_ cells	Immunotherapy	Hepatocellular carcinoma	140
**Inorganic material**	CS-metformin@MnO_2_	pH-responsive release; downregulating PD-L1 expression; promoting wound healing	--	Breast cancer	141
	HMMDN-Met@PM	GSH and pH responsiveness; peptide-mediated M2-like TAM targeting; repolarization of TAMs to an M1 phenotype; MR imaging	--	Breast cancer	142
	NMC + NTC	Aptamer and magnetics-mediated tumor targeting; downregulating the expression of HK2 and PD-L1; inhibition of glycolysis; alleviating hypoxia; dual modality imaging	PTT; PDT and Immunotherapy	Hepatocellular carcinoma	143
	Met@BF	pH-responsive charge reversal; downregulation of PD-L1 expression; inducing DC maturation	CDT	Melanoma	144
	Mn-MSN@Met-M NPs	GSH and pH responsiveness; homologous tumor cell targeting; stabilizing the STING protein and activating the cGAS-STING pathway	Immunotherapy	Lung cancer	145

ACSS1: Acyl-CoA synthetase short chain family member 1; CAR-T: chimeric antigen receptor T cell; cGAS-STING: cyclic guanosine monophosphate-adenosine monophosphate synthase-stimulator of interferon genes; PD-L1: programmed cell death 1 ligand 1; DC: dendritic cell; CDT: chemodynamic therapy; FAO: fatty acid oxidation; PTT: photothermal therapy; MMP-2: matrix metalloproteinase-2; PDT: photodynamic therapy; GSH: glutathione; TAM: tumor-associated macrophage; CTCs: circulating tumor cells; HK2: hexokinase 2; OXPHOS: oxidative phosphorylation; MDSC: myeloid-derived suppressor cell; T_reg_: regulatory T cell.

## Abbreviations

TME: tumor microenvironment
T2DM: type 2 diabetes mellitus
AMPK: adenosine monophosphate-activated protein kinase
GLP1: glucagon-like peptide 1
ECM: extracellular matrix
TAM: tumor-associated macrophage
CAFs: cancer-associated fibroblasts
CAR-T: chimeric antigen receptor T cell
MDSC: myeloid-derived suppressor cell
T_reg_: regulatory T cell
T_eff_: effector T cell
T_m_: memory T cell
CTLs: cytotoxic T lymphocytes
DC: dendritic cell
EPR: enhanced permeability and retention
CDT: chemodynamic therapy
PTT: photothermal therapy
PDT: photodynamic therapy
ICD: immunogenic cell death
ROS: reactive oxygen species
DAMPs: damage-associated molecular patterns
CRT: calreticulin
HMGB1: high mobility group box 1
LKB1: liver kinase B1
mTOR: mechanistic target of rapamycin
eIF4E: eukaryotic initiation factor 4E
S6K: ribosomal protein S6 kinase
HIF-1α: hypoxia inducible factor-1α
SREBP1: sterol regulatory element-binding protein 1
NF-κB: nuclear factor kappa-B
Eomes: eomesodermin
CaMKK2: calcium/calmodulin-dependent kinase kinase
PGC-1α: peroxisome proliferator activated receptor γ coactivator-1α
Foxp3: forkhead box p3
STAT3: signal transducer and activator of transcription 3
RFS: recurrence-free survival
OS: overall survival
CSS: cancer specific survival
DFS: disease free survival
OXPHOS: oxidative phosphorylation
HK2: hexokinase 2
FAS: fatty acid synthesis
FAO: fatty acid oxidation
GLUT1: glucose transporter 1
HMGCR: HMG-CoA reductase
ACC: acetyl-coA carboxylase
ACSS: acyl-coA synthetase short-chain family
CPT: carnitine palmitoyltransferase
PD-L1: programmed cell death 1 ligand 1
PD-1: programmed cell death 1
MMP-2: matrix metalloproteinase-2
GSH: glutathione
cGAS-STING: cyclic guanosine monophosphate-adenosine monophosphate synthase-stimulator of interferon genes
CTCs: circulating tumor cells
